# Statistical complexity is maximized in a small-world brain

**DOI:** 10.1371/journal.pone.0183918

**Published:** 2017-08-29

**Authors:** Teck Liang Tan, Siew Ann Cheong

**Affiliations:** 1 Division of Physics and Applied Physics, School of Physical and Mathematical Sciences, Nanyang Technological University, 21 Nanyang Link, Singapore 637371, Republic of Singapore; 2 Complexity Institute, Nanyang Technological University, Block 2 Innovation Centre, Level 2 Unit 245, 18 Nanyang Drive, Singapore 637723, Republic of Singapore; Lanzhou University of Technology, CHINA

## Abstract

In this paper, we study a network of Izhikevich neurons to explore what it means for a brain to be at the edge of chaos. To do so, we first constructed the phase diagram of a single Izhikevich excitatory neuron, and identified a small region of the parameter space where we find a large number of phase boundaries to serve as our edge of chaos. We then couple the outputs of these neurons directly to the parameters of other neurons, so that the neuron dynamics can drive transitions from one phase to another on an artificial energy landscape. Finally, we measure the statistical complexity of the parameter time series, while the network is tuned from a regular network to a random network using the Watts-Strogatz rewiring algorithm. We find that the statistical complexity of the parameter dynamics is maximized when the neuron network is most small-world-like. Our results suggest that the small-world architecture of neuron connections in brains is not accidental, but may be related to the information processing that they do.

## Introduction

The brain, often compared to the central processing unit (CPU) of a digital computer, handles and processes information from all our sensory organs. We clearly benefit from it’s capability to hold thoughts and conciousness, even though the working principles of the brain is not yet fully understood. While neuron level dynamics has been thoroughly studied [1], and extensive work has been done to map the functional regions [2], we do not have an established theory connecting functional regions to information processing at the whole-brain level. Unlike a digital computer, the brain is complex, and its information processing capabilities might be an emergent property [3]. Indeed, the brain is suggested to be critical—with power laws found, first by Bak et al. in their learning model [4], then by Beggs in neurons grown on petri dishes [5], and lastly by Kitzbichler et al. in vivo in a functioning human brain [6]. These discoveries prompted science journalist to claim that “the human brain is on the edge of chaos” [7, 8].

Chialvo further argued that the emergent complex neural dynamics in brains are essential to “navigate a complex, critical world” [3]. The logic behind the argument is that critical systems can display long-range (power-law) correlations in space and time, and therefore it is very important to allow neural networks that process information from such systems to have neuron-neuron interactions that go beyond immediate neighbours. Indeed, computer scientists have shown that the computational capability of a neural network is maximised at the edge of chaos [9, 10], so that it is able to assign unique non-random outputs to the most number of inputs. Crutchfield and Young earlier developed a measure of statistical complexity based on his *ϵ*-machines quantifying the information density of complex patterns [11, 12], and found that the complexity of a dynamical system is maximized at the edge of chaos. At the same time, others looking into brain-related networks discovered that they have small-world properties [13–15]. Is this network topology unrelated to the edge of chaos, or is it a consequence of the brain being at the edge of chaos? Our goal is to understand the brain more deeply by linking all these parallel concepts to ask broader questions as what it means for the brain to be at the edge of chaos? Why is it at the edge of chaos? How does it get the edge of chaos? What can it do at the edge of chaos? Specifically in this paper, we check whether complexity is indeed maximized for a small-world brain.

We organised our paper into five sections. In the Neuron Model section, we explain the need to pick a biologically realistic neuron model. This is because we are interested in emergent phenomenon, therefore we should not use models at the functional or whole-brain level. In the literature, the Hodgkin–Huxley model [16] is the most realistic, but it is as the same time the most computationally expensive. On the other hand, integrate-and-fire models [17] are computationally cheap, but they are not realistic. We strike a compromise by using Izhikevich’s model [18], which balances realism with computational tractability. We then map out the detailed phase diagram of a single neuron which lead us to discover a narrow region of the phase diagram that is reminiscent of the edge of chaos. In the Parametric Coupling section, we test whether it is possible to have neurons ‘kick’ each other from one phase to another in this narrow region of the phase diagram, by parametrically coupling neurons together in an artificial energy landscape. We found that the neurons eventually relaxed to a distribution consistent with equilibrium thermodynamics. In the Complexity and Small-World Network section, we measure the transient dynamics of various small-world networks of neurons, and found that the measured statistical complexity of their dynamics peaks close to where the network is most small-world-like, as characterised by the gap between the average clustering coefficient and the average path length.

## Neuron model

Integrate-and-fire models [17] introduced as early as 1907 and their variants [19, 20] were the first ever models used to mimic the dynamics of a neuron. These models were highly popular at first as they capture the essence of neuron firing behaviour without detailed consideration of the biological mechanisms, and at a very low computational cost. On the other hand, the Hodgkin-Huxley model [16], introduced in 1952 was hailed as the first biologically inspired model that captures not only the overall dynamics of the neuron but also detailed the internal mechanism. Several other biologically inspired models were developed subsequently [21] aiming to reduce computational cost. We eventually chose the computationally lightweight Izhikevich’s model in which different neuron types corresponds to different parameters (see Ref. [18]).

### Izhikevich’s simplified neuron model

In his 2003 paper, Izhikevich demonstrated that his model was able to achieve both “biologically plausibility of Hodgkin–Huxley-type dynamics and the computational efficiency of integrate-and-fire neurons” [18]. He achieved this by using bifurcation methodologies to map the biophysically accurate Hodgkin–Huxley-type neuronal models to a two-dimensional system of ordinary differential equations of the form [22]

$$\begin{array}{c} \dot{v}=0.04v^{2}+5v+140-u+I \end{array}$$ (1)

$$\begin{array}{c} \dot{u}=a\left(bv-u\right) \end{array}$$ (2)

with the auxiliary after-spike resetting

$$\begin{array}{c} \mathrm{if}v\geq 30\text{mV,}\;\text{then}\;\{\begin{array}{c} v\leftarrow c\\ u\leftarrow u+d. \end{array} \end{array}$$ (3)

Here, *a*, *b*, *c*, and *d* are dimensionless parameters, and the notation $\dot{v}=dv/dt$, where *t* is the time. The variables *v* and *u* are dimensionless and represent the membrane potential of the neuron and membrane recovery variable respectively. The membrane recovery variable, *u*, provides negative feedback to *v* and accounts for the activation of K^+^ ionic currents and inactivation of Na^+^ ionic currents. When the spike reaches its peak (+30 mV), Eq (3) resets *u* and *v*. Lastly, *I* represents either synaptic currents or injected direct currents.

The summary of parameters used to simulate seven different spiking types in Izhikevich’s model can be seen in Ref. [18]. The excitatory neurons are divided into three classes: RS (regular spiking), IB (intrinsically bursting), and CH (chattering), while the inhibitory neurons are divided into two classes: FS (fast spiking) and LTS (low-threshold spiking). There are two other neuron types that fall outside of the above classification scheme, and they are TC (thalamo-cortical) neurons and RZ (resonator) neurons.

### Phase diagram

Even though Izhikevich developed his simplified model relying on the fact that the spiking behaviour changes abruptly when we go from one spiking type to another, he did not sketch a phase diagram for his model. Touboul performed a rigorous analysis on parameters *I*, *a* and *b* to identify the bifurcations separating different spiking phases on the *a*-*b* parameter space [23], but not on *c* and *d* which appears in the reset conditions Eq (3). Hence, our first order of business is to fully characterize the phase diagram of a single neuron. To do this, we adopted the 4th-order Runge–Kutta method (RK4) with step size *h* = 10^−3^ to implement Eq (1) and Eq (2) following the steps:

$$\begin{array}{c} \begin{array}{cc} A_{1} & =0.04v_{n}^{2}+5v_{n}+140-u_{n}+I,\\ B_{1} & =a(bv_{n}-u_{n}),\\ A_{2} & =0.04{\left(v_{n}+hA_{1}/2\right)}^{2}+5\left(v_{n}+hA_{1}/2\right)+140-\left(u_{n}+hB_{1}/2\right)+I,\\ B_{2} & =a[b\left(v_{n}+hA_{1}/2\right)-\left(u_{n}+hB_{1}/2\right)],\\ A_{3} & =0.04{\left(v_{n}+hA_{2}/2\right)}^{2}+5\left(v_{n}+hA_{2}/2\right)+140-\left(u_{n}+hB_{2}/2\right)+I,\\ B_{3} & =a[b\left(v_{n}+hA_{2}/2\right)-\left(u_{n}+hB_{2}/2\right)],\\ A_{4} & =0.04{\left(v_{n}+hA_{3}\right)}^{2}+5\left(v_{n}+hA_{3}\right)+140-\left(u_{n}+hB_{3}\right)+I,\\ B_{4} & =a[b\left(v_{n}+hA_{3}\right)-\left(u_{n}+hB_{3}\right)],\\ v_{n+1} & =v_{n}+h\left(A_{1}+2A_{2}+2A_{3}+A_{4}\right)/6,\\ u_{n+1} & =u_{n}+h\left(B_{1}+2B_{2}+2B_{3}+B_{4}\right)/6. \end{array} \end{array}$$ (4)

The terms *A*_1_, *A*_2_, *A*_3_, *A*_4_ and *B*_1_, *B*_2_, *B*_3_, *B*_4_ are intermediates required to be computed for the variables *v*_*n*_ and *u*_*n*_ respectively. Here *v*_*n*_ and *u*_*n*_ are the membrane potential variable and the membrane recovery variable at time *t* = *nh*. Then, with the input of the initial conditions *v*_0_ and *u*_0_, together with the parameters *h*, *I*, *a*, *b*, *c* and *d* we can compute from Eq (4) the values of *v*_*n*_ and *u*_*n*_.

**Fig 1:**
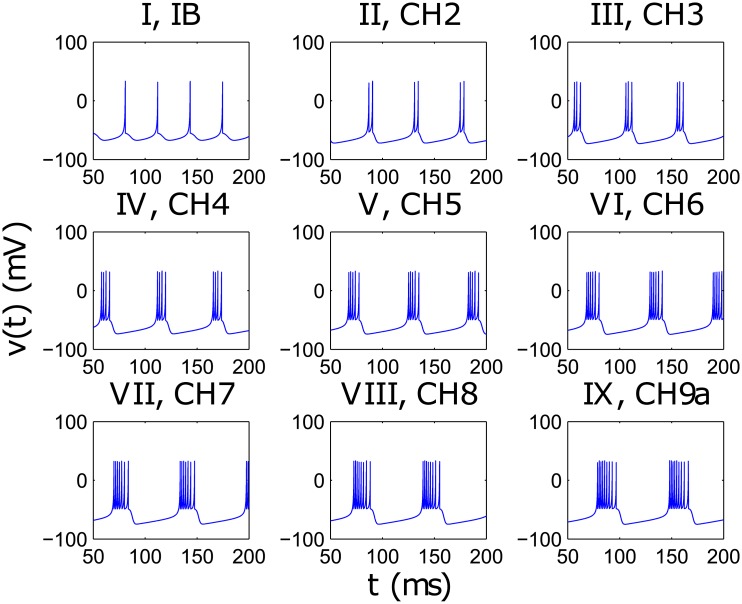
Observable differences in the time series with gradual variation of the parameters *c* and *d* from IB to CH. The nomenclature here follows the number of spikes in the spike trains observed and the Roman numbering is assigned respectively.

**Fig 2:**
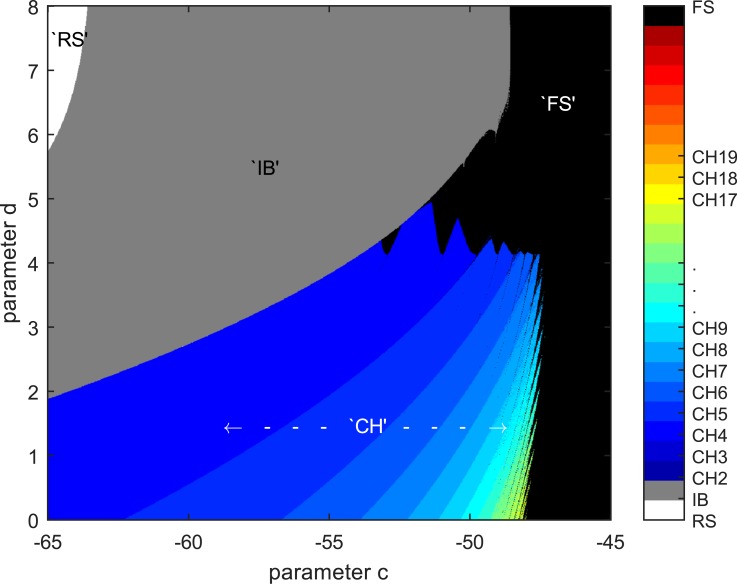
Phase diagram for parameters *c* and *d* while fixing *a* = 0.02 and *b* = 0.2, which is the common parameter values for RS, IB, and CH seen in Ref. [18]. This plot is obtained at integrating step-size of *h* = 5 × 10^−3^, and resolution of parameters *c* and *d* is 2 × 10^−2^. Each shade of colour is assigned after systematical examination of the time series of the membrane potential, *v*, in accordance to that displayed in Fig 1.

After replicating the results from Izhikevich’s model, further exploration revealed distinct changes in the time series, seen in Fig 1, as the spiking type goes from IB to CH as we vary the parameter in the *c*-*d* plane. A systematic examination of Izhikevich’s model in the *c*-*d* plane yields the phase diagram shown in Fig 2. In particular, the “rainbow” CH regime is reminiscent of the period doubling route to chaos in the logistic map, and is thus a very promising area to explore for possible encoding of information, as each “shade” enclosed one distinct spiking time series. We can treat the small region in parameter space with a proliferation of regimes before the system enters the FS regime as the edge of chaos and these different spiking regimes as the basis to build a computational representation of information.

As we have explained in the Introduction, we believe that the brain is at the edge of chaos in order to process complex information sensed from the environment. However, it is possible that the whole brain is at the edge of chaos, but none of its functional parts are. Alternatively, the brain can be at the edge of chaos at every scale. That is to say, even if we restrict ourselves to examining a small part of the brain, even this small part resides at the edge of the chaos. We do not know which scenario is true, or whether the biological brain is somewhere between these two extremes. In fact, there is no literature on the frequency distribution of neuron types in a functional brain. In other words, if we imagine we can extract the Izhikevich parameters for each and every neuron in the brain, and plot them as points in the Izhikevich parameter space, we do not know whether these points are uniformly distributed (the whole brain is at the edge of chaos, but not all its parts), or they are clustered (the brain is at the edge of chaos at multiple scales). Even though we do not have the answer to the above question, we felt we can still take a baby step forward to understanding the problem of information processing by a brain at the edge of chaos by studying a small network of CH neurons all of which are close to the edge of chaos. If we can demonstrate that a small network of CH neurons at the edge of chaos can encode information, then a larger network using similar building blocks will then be able to do more.

## Parametric coupling

In the literature, neurons are mostly pulse-coupled [24]. Some papers even suggested that learning in the brain is accomplished through changing the synaptic connection strength alone [25–28]. Neuron types plasticity is currently an experimental idea, where we find papers suggesting possible alteration through external effects like viruses [29], optical stimulations [30], and chemicals [31]. In simulation studies of neuron-neuron interactions, plasticity in neurons types have not been thoroughly explored. It appears therefore that the neuroscience community is starting to explore this phenomenon more seriously, in particular to elucidate the mechanism(s) behind the change in excitability of neurons [32, 33]. Whatever the nature of non-synaptic neuron plasticity, at the level of Izhikevich’s model it must be mediated by terms where the output of a neuron is directly coupled to the parameters of another neuron, even if the mechanism involve synaptic processes. As we can see, the phase diagram in Fig 2 suggests an alternative adaptation process that can have direct implications on information processing if neuron types can change. Therefore, in addition to pulse coupling, we also introduce parametric coupling, where the parameters of one neuron are affected by the outputs of the neurons it is connected to.

In principle, the spiking activities of the neighbors of a neuron change the local electrostatic and chemical environments it is in. This dynamic environment can push the neuron from one spiking type to another, provided its parameters are close to the phase boundary between the two spiking types. Unfortunately, a simulation at this level of detail would be prohibitively expensive and complicated. Therefore, as a test of concept we adopt a design approach based on an artificial energy landscape. In this framework, we treat the neuron parameter *Q* as a particle moving in a *phase-diagram-inspired energy landscape*, *E*, so that it will experience a force *F*_*e*_ due the to potential gradient driving it to the nearest local minimum. The particle also experiences a friction force *F*_*r*_ when moving within this artificial energy landscape. Finally, the particle are connected to other particles through springs, so that they will exert spring forces *F*_*s*_ on each other. With the aid of Verlet integration, we can write these equations of motion mathematically as

$$Q_{n+1}=2Q_{n}-Q_{n-1}+Ah^{2},$$ (5)

$$A=A\left(Q_{n}\right)=\left(F_{e}+F_{s}+F_{r}\right)/m,$$ (6)

$$F_{s}=-K_{s}\sum_{i\neq j}^{neighbours}(Q^{\left(i\right)}-Q^{\left(j\right)})\theta (v^{\left(j\right)}),$$ (7)

$$F_{r}=-K_{r}\left(Q_{n}-Q_{n-1}\right)/h,$$ (8)

$$F_{e}=\frac{dE(Q_{n})}{dQ_{n}}.$$ (9)

In the above equations, *Q* parametrizes the straight line going from (*c*, *d*) = (−55, 4) to (*c*, *d*) = (−50, 2). This effectively reduced the parameters values in Fig 1 to *Q* = −2 for RS, *Q* = 0 for IB, and *Q* = 1 for CH. Here, *Q*_*n*_ are the values of *Q* at time step *t* = *n* ⋅ *h*, while *h* = 10^−3^ is the integration step size, *K*_*s*_ = 25 is the spring constant, and *K*_*r*_ = 1 is the friction coefficient. The superscripts in Eq (7) refers to the index of the neuron node. The Heaviside step function *θ*(*v*^(*j*)^) term in Eq (7) ensures the update of *Q*^(*i*)^ occurs only when its neighbour neuron *j* is spiking.

### Phase-diagram-inspired energy landscape

**Fig 3:**
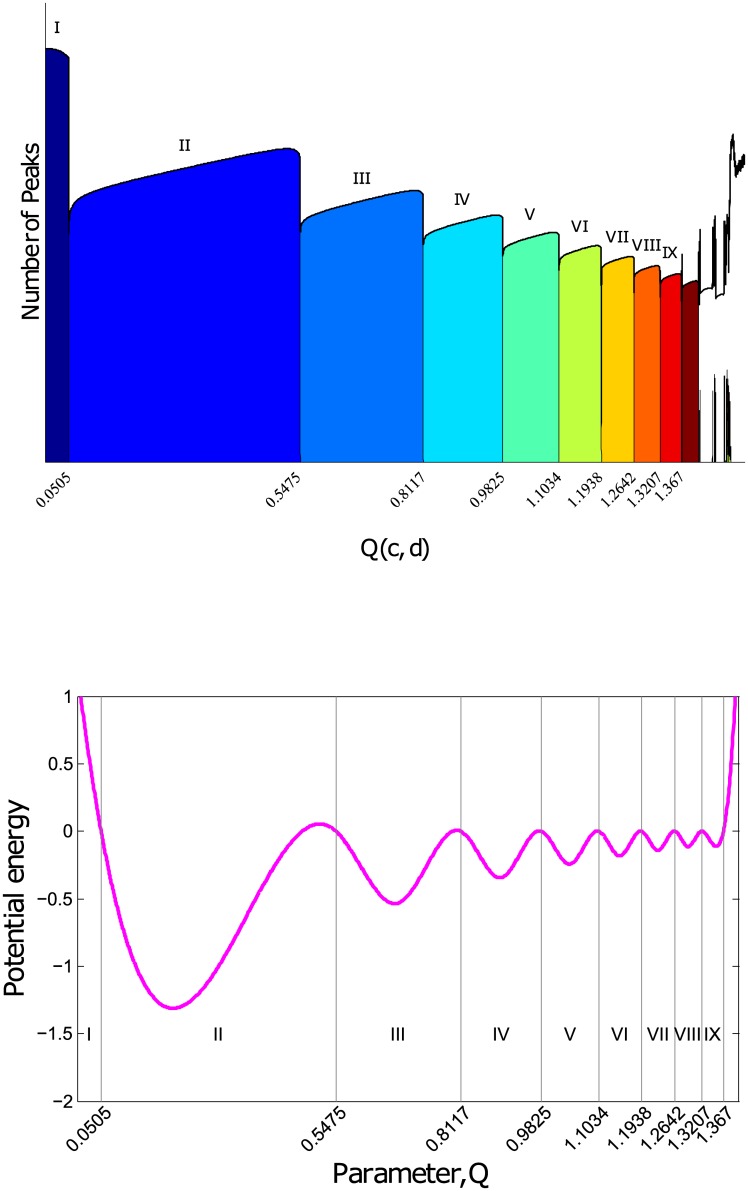
*Top*: Boundaries of each of the various spiking regimes found by collecting long times series of each of the parameter values from *Q* = 0 to *Q* = 2 (showing only *Q* = 0 to *Q* = 1.4 because beyond this value of *Q*, the neuron is in the FS regime) in steps of 4 × 10^−5^ and using peak analysing technique to segregate and count the various peak type observed. The vertical axis represents the number of peaks per unit time, and spiking types are labelled in Roman numbering as illustrated in Fig 1. *Bottom*: Phase Diagram Inspired Energy Landscape obtained by performing a cubic spline of critical turning points of the boundaries and their midpoints. The absolute value of the energies is inconsequential to the dynamics, thus the energy at the boundaries is arbitrarily set to 0 while that of their midpoints is set to the difference of the parameter values at the boundary.

To construct an artificial energy landscape with a ‘thermodynamic’ equilibrium that is consistent with the phase diagram, we determine the sharp regime boundaries from the various spiking types displayed in Fig 1 and the counting of number of peaks per unit time in Fig 3(*top*), and assign these as energy value of zero in the artificial energy landscape. We then assign a negative energy value whose magnitude is proportional to the width of the spiking regime at the middle of each spiking regime. Once these two sets of points on the artificial energy landscape have been identified, we fit a cubic spline through them to obtain the energy landscape as shown in Fig 3(*bottom*).

While one may realized that it is also possible to simulate parametric dynamics over the full (*c*, *d*) parameter space. This would be a lot more involved numerically compared to what we have done in this paper. More importantly, an energy landscape over the full (*c*, *d*) parameter space would consists of multiple elongated flat directions. A neuron anywhere along a flat direction would be in the same CH*n* spiking phase, so the most important aspect of its parametric dynamics would be that normal to the phase boundary. Fortunately, when we chose to restrict our simulations to the straight line parametrized by *Q*, this straight line is very close to being normal to the set of CH*n* phase boundaries. Ultimately, if we want to be strictly normal to all phase boundaries, we would need to restrict our simulations to be along a curve, which would again be more troublesome.

### Quasi-equilibrium distribution of parameters

**Fig 4:**
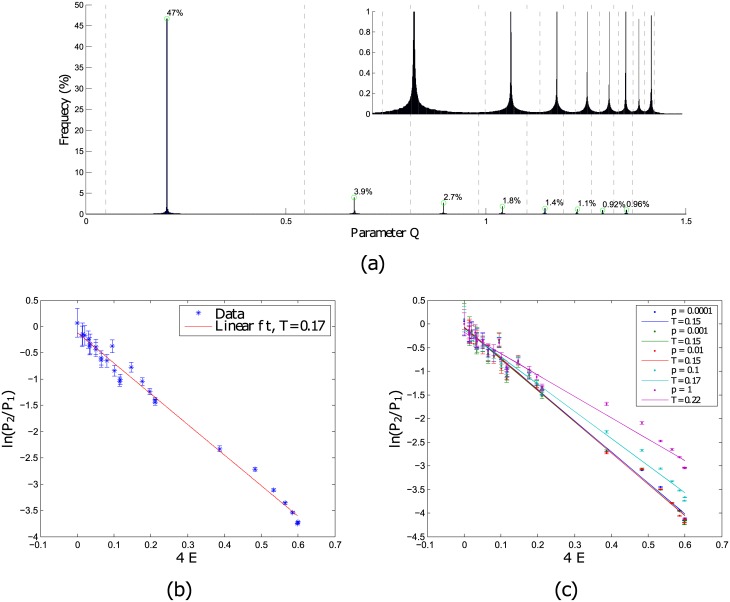
(a) Frequency distribution of parameters with the gray dotted line marking the transition boundaries of parameters seen in (Fig 3). This data is collected over 40 sets of network of size, *N* = 200, and number nearest neighbour, *k* = 3. The inset on the top right is the zoom-in showing the spread of the parameters about the peaks of their distribution. (b) Ratio of probabilities of parameters in each energy well against the energy differences of the wells. (c) Segregated by different network rewiring probabilities, p.

To perform a computation, we need to first be able to represent different information. In a digital computer, this is done by using strings of ‘1’s and ‘0’s, representing the on and off states of logic gates. In principle, other forms of representation can also work. For example, the distinct spiking regimes in the ‘edge of chaos’ of the Izhikevich neuron model can be used as a representation. To use such a basis, the spike type must be able to switch from one to another during the course of a computation. Indeed, when the parameters of our coupled neuron system were allowed to vary in the energy landscape, we achieved a quasi-equilibrium distribution of the parameters, as shown in Fig 4.

In this quasi-equilibrium distribution, we can estimate the temperature of the system using the ratio of probabilities of parameters in each energy well and the energy differences of the wells in the thermodynamic relation, $\frac{P_{1}}{P_{2}}\propto \exp(-\frac{\Delta E}{T})$. Starting with a ring network of neurons, we find that with increasing rewiring probability, the temperature of the system increases, as seen in Fig 4(c). This means that the system can easily switch from one spike type to another spike type. However, an infinite temperature is not be desirable here, as that would quickly randomize the information.

## Complexity and small-world network

Like many other brain scientists, we believe that information encoding in the brain is not done at the level of single neurons, but over a network of neurons [3]. Hence, the final missing ingredient to understanding information encoding in the brain is the topology of the neuron network. For this final part of the study, we chose to work with small-world networks. This network topology allows a neuron to trigger another neuron far away and is even capable of self-sustaining activity [34–36]. Moreover, many papers have also found that the small-world property is a prominent common ingredient in the functional network of the human brain [13–15]. In particularly, Sporns et al. found by generating a family of networks that the complexity of the static network topology is maximized in small-world-like networks, and even more so in network topologies derived from actual neuroanatomical data [37].

**Fig 5:**
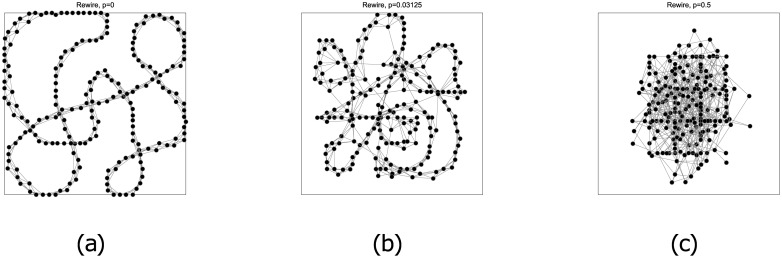
Three networks with *N* = 200 nodes each connected to the nearest *k* = 3 neighbours with increasing rewiring probabilities, (a) *p* = 0 (regular ring), (b) *p* = 1/32 (small-world), and (c) *p* = 1/2 (random).

To set-up our small-world network, we used the Watts-Strogatz [38] *rewiring algorithm* to generate networks with *N* = 200 nodes, each connected to 2*k* other nodes. This algorithm allows us to continuously tune the network from a regular ring network (with *N* nodes each connected to the nearest *k* neighbours) to a fully random network, by adjusting the probability, *p*, of rewiring the connection (see Fig 5). Newman and Watts also proposed an alternative way to link distant nodes, by starting from the regular network, and adding shortcuts between randomly chosen pairs of nodes [39]. We call this the *shortcut algorithm*.

### Complexity measures

The hypothesis we would like to test in this paper is that the brain is in a self-organized critical state, within which it has a large complexity, and also a small-world network topology. Self-organised criticality (SOC), proposed by Per Bak in 1987 [40, 41], is a concept closely intertwined with complexity [42]. However, while the concept of SOC is well established and clearly defined [40, 41, 43], that of complexity is generally understood but lacks unanimous definition [44]. Many have offered methods to compute or estimate the complexities of dynamical systems [12, 45]. In particular, using the *ϵ*-machine representation of a process, Crutchfield showed that complexity is maximised in dynamical systems as they approach the edge of chaos [12, 46].

With this in mind, we seek to find the parallel phenomenon of complexity peaking between order and disorder in our computational model of neuron dynamics. However, instead of measuring Crutchfield’s *ϵ*-machine complexity, we measured the statistical complexity *C*_*LMC*_ introduced by Lopez-Ruiz, Mancini, and Calbet [45]. This *C*_*LMC*_ complexity measure is a good estimate of complexity based on a probabilistic description of the dynamics, and is also computationally lightweight in comparison to James Clutchfield’s *ϵ*-machine.

**Fig 6:**
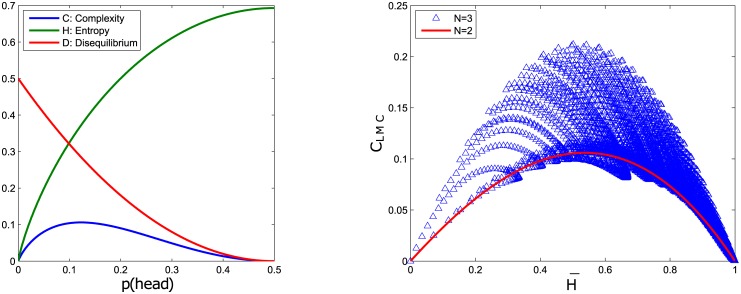
*Left*: Plot of the statistical complexity *C*_*LMC*_, entropy *H* and disequilibrium *D* versus the probability of obtaining a head outcome in a biased coin (*N* = 2 states) system. To calculating *C*_*LMC*_ in a 2-state system we can simply rewrite Eqs (11) and (12) as *H* = −[*p*(*head*)log *p*(*head*) + *p*(*tail*)log *p*(*tail*)] and *D* = {[*p*(*head*) − 1/2]^2^ + [*p*(*tail*) − 1/2]^2^}. Since we have *p*(*head*) = 1 − *p*(*tail*), the graph is symmetrical about *p*(*head*) = 0.5. *Right*: Plot of the statistical complexity *C*_*LMC*_ versus the normalised entropy $\bar{H}$ for *N* = 2 and *N* = 3 states systems.

For a system with *N* accessible states *x*_1_, *x*_2_, …, *x*_*N*_ each with corresponding probabilities *p*_1_, *p*_2_, …, *p*_*N*_, the *C*_*LMC*_ complexity is defined as

$$\begin{array}{c} C_{LMC}=H\cdot D, \end{array}$$ (10)

where

$$\begin{array}{c} H=-\sum_{i=1}^{N}p_{i}\log p_{i} \end{array}$$ (11)

is the Shannon entropy, a measure of disorderedness, and

$$\begin{array}{c} D=\sum_{i=1}^{N}{\left(p_{i}-1/N\right)}^{2} \end{array}$$ (12)

is named as the *disequilibrium*. With this definition we have the complexity *C*_*LMC*_ = 0 in both a fully ordered system where the entropy *H* = 0, and in a equilibrium system where the probabilities of the states are uniformly random *D* = 0 despite having high entropy *H*. For any other system the complexity *C*_*LMC*_ will have a value higher than zero which represents that the system is in neither complete order nor in equilibrium (see Fig 6 *Left* for more details on the calculations of *C*_*LMC*_). The relationship between *C*_*LMC*_ and *H* gets complicated quickly with larger systems (*N* > 2). Fig 6 *Right* illustrate a *N* = 3 states system where multiple combinations of probabilities *p*_*i*_ results in the same magnitude of entropy *H* yet different complexities *C*_*LMC*_. However the fact remains that *C*_*LMC*_ does not grow indefinitely with entropy *H*.

### Maximising of complexity

The key network parameters to define a small-world network is the local clustering coefficient *C* measuring the cliquishness of a typical neighbourhood and characteristic path length *L* measuring the typical separation between two vertices in the graph [38]. More concretely, *C*_*i*_ of a particular *i*^*th*^-node is calculated by the proportion of links between the vertices within its neighbourhood divided by the number of links that could possibly exist between them, hence the network average clustering coefficient is $C\left(p\right)=\frac{1}{N}\sum_{i=1}^{N}C_{i}$ where *p* is the rewiring probability tuning the network topology and *N* is the total number of nodes. As for the characteristic path length we have $L\left(p\right)=\frac{1}{N(N-1)}\sum_{i\neq j}d\left(i,j\right)$ where *d*(*i*, *j*) is the shortest distance between *i*^*th*^- and *j*^*th*^-node.

**Fig 7:**
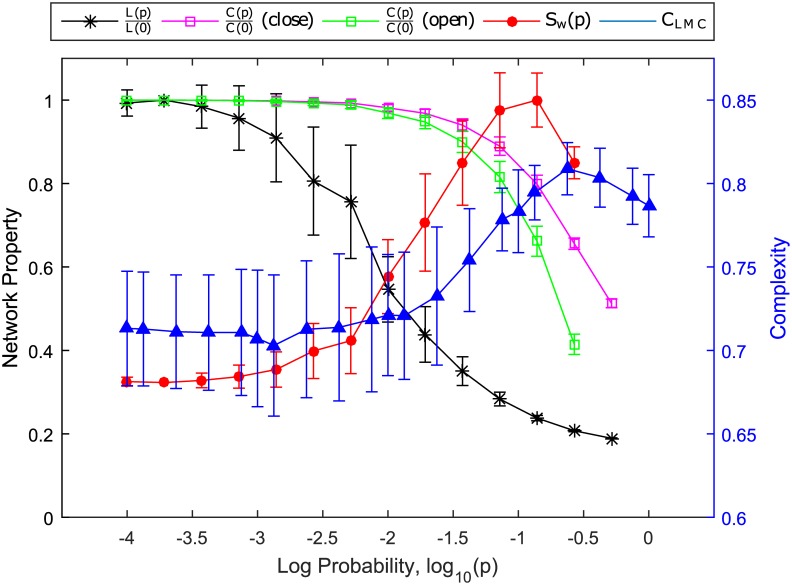
*Left axis*: Plot of clustering coefficient *C*(*p*)/*C*(0) and characteristic path length *L*(*p*)/*L*(0) of the networks, both scaled so that their maxima are one, with varying rewiring probabilities, *p*. The ratio between these two properties, *S*_*w*_(*p*) = *C*(*p*)/*L*(*p*), is indicative of the transition to a small world network. In this figure, *S*_*w*_(*p*) is also scaled so that its maximum is one. *Right axis*: Plot of the statistical complexity *C*_*LMC*_ versus the rewiring probability *p*, averaged over the parameter time series *Q*^(*i*)^(*t*) of the *i* = 1, …, 200 neurons. We do not show the clustering coefficient *C*(*p*) versus varying probability *p* to add a shortcut because the original *k*-nearest-neighbor network was not destroyed by adding shortcuts, even at very high *p*, and thus the resulting network is not fully random. *Note: Connecting lines were added the data points only to enhance visibility of the trends*.

As we tune the *N* = 200 and *k* = 3 network by increasing the rewiring probability *p*, we find in Fig 7 that the characteristic path length *L*(*p*) decreases rapidly, whereas the average clustering coefficient *C*(*p*) remains large and only starts its rapid decrease after *p* becomes large enough. The network is close to being regular when *L*(*p*) and *C*(*p*) are both large, and is close to being random when *L*(*p*) and *C*(*p*) are both small. In between these two extremes, the network has small *L*(*p*), but large *C*(*p*), and is manifestly small-world. Therefore, the ratio *S*_*w*_(*p*) = *C*(*p*)/*L*(*p*) has a peak at intermediate rewiring probability *p*. When we apply the shortcut algorithm, *L*(*p*) has the same behavior as the probability *p* of adding a shortcut increases. However, *C*(*p*) remains large even at large *p*, so there is no well-defined peak in *S*_*w*_(*p*).

The calculation of the *C*_*LMC*_ statistical complexity is obtained first by reducing the dynamics of *Q*^(*i*)^(*t*) to its symbolic dynamics of the nine CH regimes (indicated by Roman numerals in Fig 1. This is done by computing the averages of *Q*^(*i*)^(*t*) over non-overlapping time windows of 50 time steps each. Since one time step is equivalent to 1 × 10^−3^ time unit, the size of each time window is equivalent to Δ*t* = 0.05. Secondly, a scale of 4 is chosen to analyse as states of the system resulting in a total of 9^4^ possible states, which is comparable to using a scale of 12 for $x<\frac{1}{2}$ and $x>\frac{1}{2}$ in the logistic map, i.e. 2^12^ states [45]. Lastly, we collect the symbolic time series of all *N* = 200 neurons over the 10,000 time windows, and over 40 different initial conditions, to determine the probabilities of each of the 9^4^ symbolic state. With this probability distribution, we calculate the values of *H*, *D* and *C* using Eqs (10) to (12).

Moving from ordered ring network to random network, we demonstrated in Fig 7 that the statistical complexity *C*_*LMC*_(*p*) of a network of parameter-coupled neurons evolving within a phase diagram inspired energy landscape peaks close to the peak of *S*_*w*_(*p*). In other words, the peak in statistical complexity coincides with the peak in small-world character of the neuron network. This result suggests that for the brain to maximize its statistical complexity at the edge of chaos for effective information processing, it should also have the strongest small-world character.

## Conclusions

In this paper, we explored the plausibility of information processing in brains that are at the edge of chaos, and how this information processing is related to the empirically observed small-world topology of brain functional networks. We do this by identify the CH region in the phase diagram of a Izhikevich neuron, that has the characteristics of being at the edge of chaos, i.e. many distinct CH regimes are accessible within a small parameter region. We then couple Izhikevich neurons such that the output of a neuron changes the parameters of neurons it is coupled to. We called this *parametric coupling*, and simulated the dynamics of the set of neuron parameters on a phase diagram inspired energy landscape.

Using the rewiring algorithm of Watts and Strogatz, we interpolate between regular neuron networks, small-world neuron networks, and random neuron networks, and find that the statistical complexity of the neuron dynamics peaks when the small-world character of the network also peaks. This suggests that the small-world character of brains is connected to the statistical complexity of brains sitting at the edge of chaos.

With this proof of concept, we now have the foundation to move the study forward to understanding how information can be actually encoded and processed within such neuron networks. We would then move on to identifying possible built-in logics gates in the network that can manipulate information. These are small but over-represented dynamical motifs that appear in brain information processing. With these, we aim to better understand the computational capability of the brain at the edge of chaos. We believe that in science, it is not only important to obtain results, but also to ask the right questions that would frame the problem. What it means for the brain to be at the edge of chaos? Why is it at the edge of chaos? How does it get the edge of chaos? What can it do at the edge of chaos? The entire set of questions constitutes an ambitious research program that would take much time to be completed. The results we present in this paper partially answers some of these questions, and should be appreciated in this light.

## Supporting information

S1 Dataset Final results dataset. Dataset of the final results used to plot the graphs in this paper.
(ZIP)
